# The role of iron in the pathogenesis of endometriosis: a systematic review

**DOI:** 10.1093/hropen/hoad033

**Published:** 2023-07-27

**Authors:** James Wyatt, Sean M Fernando, Simon George Powell, Christopher J Hill, Ilyas Arshad, Chris Probert, Shakil Ahmed, Dharani K Hapangama

**Affiliations:** Department of Women’s and Children’s Health, Institute of Life Course and Medical Sciences, University of Liverpool, Liverpool, UK; Liverpool University Hospitals NHS Foundation Trust, Liverpool, UK; School of Clinical Medicine, University of Cambridge, Cambridge, UK; Department of Women’s and Children’s Health, Institute of Life Course and Medical Sciences, University of Liverpool, Liverpool, UK; Liverpool University Hospitals NHS Foundation Trust, Liverpool, UK; Department of Women’s and Children’s Health, Institute of Life Course and Medical Sciences, University of Liverpool, Liverpool, UK; Liverpool Women’s Hospital NHS Foundation Trust, Liverpool, UK; Liverpool University Hospitals NHS Foundation Trust, Liverpool, UK; Department of Molecular and Clinical Cancer Medicine, Institute of Systems, Molecular and Integrative Biology, University of Liverpool, Liverpool, UK; Liverpool University Hospitals NHS Foundation Trust, Liverpool, UK; Department of Women’s and Children’s Health, Institute of Life Course and Medical Sciences, University of Liverpool, Liverpool, UK; Liverpool Women’s Hospital NHS Foundation Trust, Liverpool, UK

**Keywords:** endometriosis, ferroptosis, iron, iron chelation, iron excess, oxidative stress, systematic review

## Abstract

**STUDY QUESTION:**

What is the role of iron in the pathophysiology of endometriosis?

**SUMMARY ANSWER:**

Iron excess is demonstrated wherever endometriotic tissues are found and is associated with oxidative stress, an inflammatory micro-environment, and cell damage; the iron-mediated oxidative stress is independently linked to subfertility, symptom severity, and malignant transformation.

**WHAT IS KNOWN ALREADY:**

Iron is found in excess in endometriotic tissues, and multiple mechanisms have been studied and posited to explain this. It is clear that iron excess plays a vital role in promoting oxidative stress and cell damage. The evidence base is large, but no comprehensive reviews exist to summarize our understanding and highlight the overarching themes to further our understanding and suggest future directions of study for the field.

**STUDY DESIGN, SIZE, DURATION:**

This systematic review with a thematic analysis retrieved studies from the PubMed, Embase, Web of Science, and Cochrane Library databases and searches were conducted from inception through to August 2022. Human and animal studies published in the English language were included and identified using a combination of exploded MeSH terms (‘Iron’ and ‘Endometriosis’) and free-text search terms (‘Iron’, ‘Ferric’, ‘Ferrous’, ‘Endometriosis’, ‘Endometrioma’).

**PARTICIPANTS/MATERIALS, SETTING, METHODS:**

This review was reported in accordance with the PRISMA guidelines. All studies reporting original data concerning the role of iron or iron complexes in the pathophysiology of endometriosis were included. Studies that did not report original data or provided a review of the field were excluded. Bias analysis was completed for each included study by using the Newcastle–Ottawa scoring system.

**MAIN RESULTS AND THE ROLE OF CHANCE:**

There were 776 records identified and these were screened down to 53 studies which met the eligibility criteria, including 6 animal and 47 human studies, with 3556 individual participants. Iron excess is demonstrated in various tissues and fluids, including ovarian endometriomas, ovarian follicles, ectopic endometriotic lesions, and peritoneal fluid. Markers of oxidative stress are strongly associated with high iron levels, and aberrant expression of iron-transport proteins has been demonstrated. Abnormal resistance to ferroptosis is likely. Iron-mediated oxidative stress is responsible for a pro-inflammatory micro-environment and is linked to subfertility, symptom severity, and, possibly, malignant transformation.

**LIMITATIONS, REASONS FOR CAUTION:**

A minority of the included studies were of objectively low quality with a high risk of bias and may lead to misleading conclusions. Additionally, multiple studies failed to appropriately characterize the included patients by known confounding variables, such as menstrual cycle phase, which may introduce bias to the findings.

**WIDER IMPLICATIONS OF THE FINDINGS:**

Current literature depicts a central role of aberrant iron mechanics and subsequent oxidative stress in endometriosis. It is likely that iron excess is at least partly responsible for the persistence and proliferation of ectopic endometriotic lesions. As such, iron mechanics represent an attractive target for novel therapeutics, including iron chelators or effectors of the iron-oxidative stress pathway. There are significant gaps in our current understanding, and this review highlights and recommends several topics for further research. These include the role of iron chelation, resistance to ferroptosis, the relationship between iron excess and localized hypoxia, systemic iron pathophysiology in endometriosis, and the role of oxidative stress in malignant transformation.

**STUDY FUNDING/COMPETING INTEREST(S):**

J.W. and S.G.P. are supported by clinical fellowships at Liverpool University Hospital NHS Foundation trust. No additional funding was requested or required for the completion of this work. C.J.H. is supported by a Wellbeing of Women project grant (RG2137). D.K.H. is supported by a Wellbeing of Women project grant (RG2137) and an MRC clinical research training fellowship (MR/V007238/1). The authors have no conflicts of interest to declare.

**REGISTRATION NUMBER:**

A protocol was prospectively registered with the PROSPERO database in August 2021 (CRD42021272818).

WHAT DOES THIS MEAN FOR PATIENTS? The causes of endometriosis are not yet fully understood. Previous research has shown that iron levels appear to be high in endometriosis tissues, but we do not fully understand the significance of this.

This review has gathered all the current research into the role of iron in endometriosis, to better understand what happens in patients with the disease and identify areas that need further study. The findings confirm that iron levels are abnormally high in endometriosis lesions, and this is likely due to repeated episodes of bleeding. The red blood cells then break down and the iron contained within is released. High levels of iron cause inflammation and lead to damage to the surrounding cells. High levels of iron are linked to worse symptoms and infertility.

Several methods of potentially treating endometriosis are also highlighted. Binding excess iron appears to partially treat the effects of endometriosis in animals and different methods of altering the way iron interacts with cells could lead to new treatments, but this requires further research.

## Introduction

Endometriosis is a common, chronic, gynaecological inflammatory condition affecting ∼10% of women of reproductive age (Shafrir *et al.*, 2018), equating to 1.5 million women in the UK alone (WHO, 2022). The histopathological definition of the disease centres on the establishment of extra-uterine endometrium-like tissue, primarily found in the anatomical pelvis. Typical symptoms consist of chronic pelvic pain, dysmenorrhoea, and dyspareunia, and there is a strong association with subfertility and negative psychosocial impacts (Delanerolle *et al.*, 2021). The economic productivity cost has been estimated at a loss of £8.2 billion in the UK per annum, a figure that will only have risen since its estimation in 2012 (Simoens *et al.*, 2012).

Despite the high societal and individual burden, the precise pathophysiological pathways leading to disease remain uncertain (Sourial *et al.*, 2014). Sampson’s theory of ‘retrograde menstruation and transtubal migration’ (Sampson, 1927), whereby viable fragments of physiologically-shed endometrium are deposited onto the peritoneal surface (Tempest *et al.*, 2020, 2022), probably represents only a small piece of the puzzle. Retrograde menstruation can be considered a normal physiological process, identifiable in 90% of women (Halme *et al.*, 1984). Therefore, pathways that allow the establishment and maintenance of seeded endometrium have been posited. These include altered immune, hormonal, and metabolic responses (Hapangama *et al.*, 2010; Sourial *et al.*, 2014; Zondervan *et al.*, 2018). Genetics, hormonal exposure, diet, toxins, and BMI have all been implicated as endometriosis-associated factors. The theories of coelomic metaplasia, lymphatic or vascular metastases, and neonatal uterine bleeding have also been developed to explain processes that Sampson’s theory alone cannot. The answer to the question is likely to be a complex interplay between multiple pathogenic mechanisms.

Endometriotic lesions demonstrate hormonal responses similar to healthy eutopic endometrium (Chantalat *et al.*, 2020). Ectopic lesions undergo a cycle of ovarian hormone-sensitive proliferation, haemorrhage, inflammation, and fibrosis, leading to adhesion formation and, ultimately, clinical symptoms (Reis *et al.*, 2013; Lin *et al.*, 2018). Repeated localized haemorrhage and an abnormal peritoneal response to retrograde menstruation are theorized to precipitate a cumulative deposition of erythrocytes in endometriosis (Defrère *et al.*, 2008; Allavena *et al.*, 2015; Ng *et al.*, 2020). As a critical constituent of haem and haemoglobin, iron is released during subsequent erythrocytic degradation, leading to iron excess in endometriotic tissues (Maines, 2005; Ganz and Gordon, 2016).

Aberrant iron mechanics are widely demonstrated in endometriosis and are an established pathophysiological factor. Iron is an essential element in human physiology and is required for vital mechanisms, including oxygen transport, cellular energy production, and DNA synthesis (Muñoz *et al.*, 2009). However, iron is toxic in excess. Via the formation of hydroxyl radicals, iron excess leads to oxidative stress, cellular damage, DNA dysregulation, and eventual organ dysfunction (Kohgo *et al.*, 2008). As there is no iron-specific excretion pathway, iron homeostasis is tightly regulated by multiple sophisticated mechanisms (Anderson and Frazer, 2017). Despite this, localized iron excess is common in endometriotic lesions (Defrère *et al.*, 2008; Ng *et al.*, 2020).

An oxidative–antioxidative balance exists in healthy tissues and is maintained to avoid excess oxidation and subsequent oxidative stress (Kisaoglu *et al.*, 2013). Oxidative stress is defined by free radical and reactive oxygen species (ROS)-induced lipid, protein, and DNA oxidation, a process that is cytotoxic and mutagenic (Pizzino *et al.*, 2017). Oxidative stress is prevalent in various human pathologies, including cancer development, atherosclerosis, neurological degradation, and, importantly for endometriosis, initiation, and maintenance of chronic inflammation (Pizzino *et al.*, 2017).

**Figure 1.**
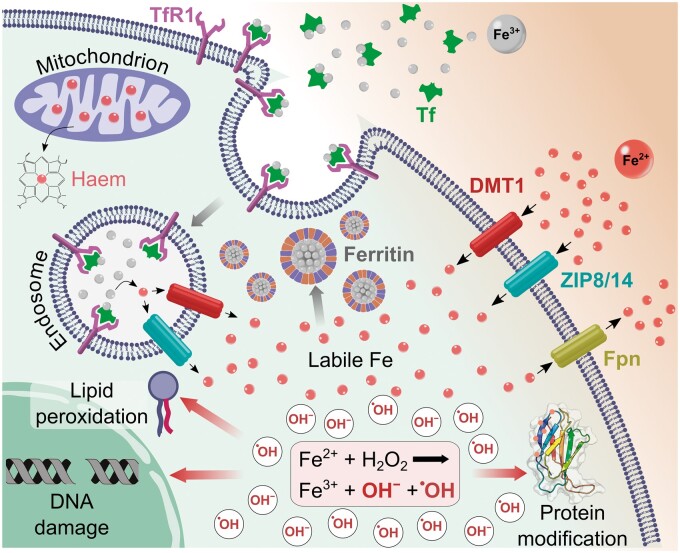
**Iron transport and homeostasis.** Schematic diagram depicts major iron transport and storage proteins. Reactive iron is capable of generating hydroxyl radicals; thus, iron accumulation increases the risk of oxidative stress. Fe^2+^, ferrous iron; Fe^3+^, ferric iron; Tf, transferrin; TfR1, transferrin receptor 1; DMT1, divalent metal transporter 1; ZIP8/14, ZRT/IRT-like protein 8 and 14; Fpn, ferroportin.

Iron exists in the ferrous (Fe^2+^) and ferric (Fe^3+^) states but can only be absorbed as ferrous iron and cannot be transported independently (Aisen *et al.*, 1999; Papanikolaou and Pantopoulos, 2005). Transferrin is the major iron-transport protein, and ferritin is the storage protein that maintains iron in a soluble, non-toxic form, mostly within the liver and bone marrow. Ferritin is composed of both H-Ferritin and L-Ferritin. H-Ferritin has a greater capacity to oxidize iron molecules and is more protective against oxidative stress. Total iron levels are a measure of iron bound to transferrin and ferritin. Free or catalytic iron represents non-transferrin-bound iron, which is highly capable of producing oxidative stress via the generation of hydroxyl radicals in the Fenton reaction (Fe^2+^ + H_2_O_2_ → Fe^3+^ + OH^−^ + OH) (Fenton, 1894; Leonard *et al.*, 2004). Haem iron refers to haem, Fe^2+^ iron bound with a protoporphyrin IX complex, an essential constituent of haemoglobin. Total iron-binding capacity (TIBC) is an indirect measure of serum transferrin levels and relates to the maximum amount of Fe^3+^ iron that a blood sample can carry. Figure 1 demonstrates the storage and transport of iron in health, in addition to the role of the Fenton reaction and its effects on the cell.

Multiple individual studies have examined iron mechanics in endometriosis but have been focused in scope and, therefore, limited in their ability to demonstrate the overall picture. Several reviews have been published on this topic but are now largely outdated, and none have been systematic in design (Defrère *et al.*, 2008; Kobayashi *et al.*, 2009; Ng *et al.*, 2020).

This review therefore aims to collate and summarize the evidence base regarding aberrant iron mechanics in endometriosis to inform readers and identify areas requiring further research.

## Materials and methods

This systematic review has been reported according to the Preferred Reporting Items for Systematic Review and Meta-Analyses (PRISMA) guidelines (Page *et al.*, 2021). A prospective protocol was registered with the International Prospective Register of Systematic Reviews (PROSPERO) database on 10 August 2021 (Registration number: CRD42021272818).

### Systematic search

A systematic search was performed using the PubMed, Embase, Web of Science, and Cochrane Library databases. All databases were searched from inception to August 2022. The search string utilized a combination of exploded MeSH terms (‘Iron’ and ‘Endometriosis’) and free-text search terms (‘Iron’, ‘Ferric’, ‘Ferrous’, ‘Endometriosis’, ‘Endometrioma’). Results were filtered to English language studies only. Grey literature was not searched.

### Eligibility criteria

#### Inclusion criteria

All human and animal studies reporting original data concerning the role of iron or iron complexes in the pathophysiology of endometriosis were included.

#### Exclusion criteria

Studies that did not report original data or provided a review of the field only were excluded. All studies without a full-text English language version were excluded. Studies not published in an established journal with a peer-review process were excluded.

### Study selection

Results from the initial searches were collated, and duplicates were deleted. Screening, data extraction, theme identification, and bias analysis were completed independently by two authors (J.W. and S.M.F.), and disagreements were resolved through discussion. The online software Rayyan (Ouzzani *et al.*, 2016) was used for the title and abstract screening.

Full texts were retrieved and assessed for inclusion using the pre-determined eligibility criteria.

Additional studies were then identified via forward and backward chaining of all studies included thus far. Similar articles, as suggested by the PubMed search engine, were also screened for inclusion. References of all relevant literature and systematic reviews identified by the initial search were also screened.

### Data extraction and synthesis

The extracted data included but were not limited to title, author, journal, year of publication, population studied, interventions, results, comparisons, and outcomes.

The results were synthesized thematically. Recurring themes were identified from the final list of included studies. Two authors (J.W. and S.M.F.) confirmed this final list of themes, which encompasses the titles presented in the results section of this review. Given the heterogeneity of the methods and results found throughout this review, no statistical meta-analysis was possible.

### Bias analysis

The Newcastle–Ottawa scale (NOS) was used to assess the quality of each study included in this review (Wells *et al.*, 2013).

## Results

### Study selection

**Figure 2.**
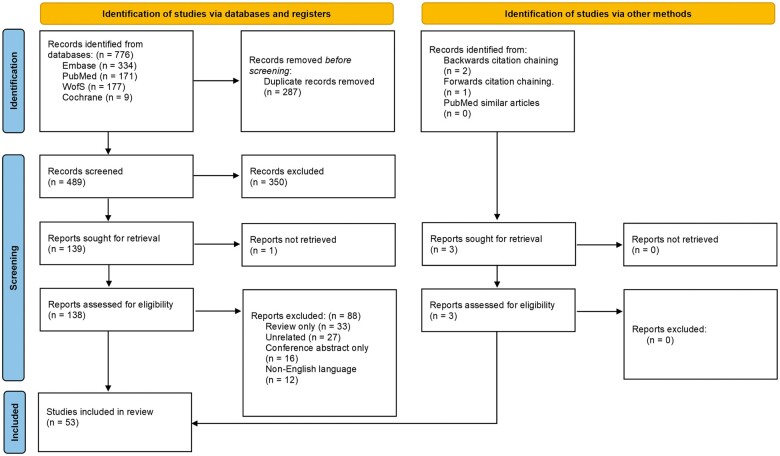
**PRISMA flow diagram.** WofS, Web of Science.

A total of 776 records were identified from database searches (Fig. 2). There were 287 duplicate records excluded, and screening excluded 350 irrelevant records. The remaining 139 studies underwent full-text review, and 89 studies were subsequently excluded as one report was not retrieved, 33 reviews of the field did not present any original data, 16 records were conference abstracts only, 27 studies were irrelevant, and 12 were not in English.

A further three studies were identified via forward and backward chaining, and all were included, providing 53 studies eligible for inclusion.

### Study characteristics

There were 6 studies that used non-human experimental models, while the remaining 47 used human bio-samples or cell lines derived from humans. A total of 3556 patients were included in the human studies. Publication dates ranged from 1994 to 2021, and various tissue types and experimental techniques were utilized (Table 1).

**Table 1.** Summary table of included studies characteristics, findings, and conclusions.

Authors (year published)	Human/animal study	n	Tissue types	Outcomes measured	Relevant findings	Conclusions drawn
Akashi *et al*. (2021)	Human	38		DMT-1, transferrin receptor, ferroportin, macrophages	Identified iron transporters, DMT1 upregulation, low TfR and fRM expression,	Malignant transformation of ovarian endometrioma
Alizadeh *et al*. (2015)	Human	70	Serum	Iron, MDA, carbonyl	High serum iron	High serum iron indicating oxidative stress
Al-Shammaa (2020)	Human	72	Serum	Hb, PCV, ESR, Serum iron	All parameters elevated in endometriosis patients on a red meat diet	Red meat diets increase serum iron
Alvarado-Díaz *et al*. (2015)	Human	10	Endometrial biopsies. Isolated endometrial stromal cells	NF-kB	Iron overload increased p65: DNA-binding activity and decreased various other expressions	Iron overload has a role in endometriosis pathogenesis and development
Alvarado-Díaz *et al*. (2016)	Human	55	Endometrial biopsies. Isolated endometrial stromal cells	Divalent metal transporter-1	Overexpression of DMT1 in endometriosis patients at various stages of menstrual cycle	Suggested a DMT1 modulated pathway of iron overload in endometriosis cases
Arumugam (1994)	Human	50	Peritoneal fluid, sperm incubated in PF	PF Iron conc. Acrosome reaction rates in sperm	Decrease in acrosome reaction rate was associated with an increase in iron content of PF	Endometriosis may play a role in infertility through acrosome reaction
Arumugam and Yip (1995)	Human	44	Peritoneal fluid	Iron levels, markers of free radical reactions	High iron levels seen in endometriosis. Correlated with disease severity	Raised iron in PF does not play a role in catalysing free radical reactions
Atkins *et al*. (2018)	Animal: Nonhuman Primate	22	Bone marrow, liver, and serum, intestinal biopsies	Iron, ferroportin	Decreased hepatic and bone marrow iron, increased ferroportin expression	Oral iron supplementation alone does not replenish iron stores in endometriosis
Bauckman *et al*. (2013)	Human	nr	Ovarian ca cell lines		DFO-induced apoptotic death in ovarian cells	Iron plays a role in modulating cell death in ovarian cancer cells
Benaglia *et al*. (2015)	Human	39	Follicular fluid from affected and contralateral ovary	Iron and ferritin. Oocyte retrieval rate	No difference seen in iron, ferritin higher in endometriosis	Iron does not play a role in ovarian function
Chen *et al*. (2021)	Animal: Mice	nr	Murine embryos	ATP level, MMP, ROS, apoptotic and ferroptotic indices	Iron exposure impaired embryo development, increased ROS, and was linked to high rates of cell death.	Iron excess in peritoneal fluid may be implicated in endometriosis-related subfertility
Chmaj-Wierzchowska *et al*. (2013)	Human	86	Serum	RANTES, CRP, Iron levels, fibrinogen, leucocytes, CA-125	No difference in serum iron or inflammatory markers	No explanation for role of inflammatory factors in endometriosis
Defrère *et al*. (2006)	Animal: Nude mice	24	Endo lesions, peritoneal fluid	Number of lesions, proliferation of lesions	Iron deposits mainly seen in macrophages and mesothelial cells	Possibility of iron chelation treatment in endometriosis
Fassbender *et al*. (2011)	Human	40	Peritoneal biopsies	Transferrin and ferritin	Reduced ferritin mRNA expression in normal peritoneum	
Guo *et al*. (2015)	Human	30	Ectopic lesions	Bilirubin, ferritin, free iron	Endometriomas of different ages varied in various features and ferritin and free iron concentration	Older endometriotic lesions have more iron content and so are wounds that undergo repeated injury and repair
Hayashi *et al*. (2020)	Animal: Mice	14		Offspring n, haemosiderin, oxidative stress, FSH	Iron accumulation reduced in endometriosis, causing oxidative stress.	Possible prevention mechanism of endometriosis
Iizuka *et al*. (1998)	Human	nr	Ovarian lesions	Iron levels	High iron levels seen in endometriosis.	Role of iron concentration assay as a diagnostic tool for evaluation of ovarian endometriotic cyst
Imanaka *et al*. (2021a)	Human	83	Cyst fluid	Total iron, haem iron	Positive concentration between dysmenorrhea severity and total and haem iron	No evidence that iron indicates severity of endometriosis-related pain. May play a role in dysmenorrhea
Imanaka *et al*. (2021b)	Human	307		Total iron, haem iron, free iron. Relationship with MRI	R2 value correlated with iron levels	MR relaxometry may be a better alternative to CF iron test in diagnosing OMA
Imanaka *et al*. (2021c)	Human	236		Total iron, haem iron, free iron, oxuHb, 8-OHdG, metHb, antioxidants, TAC	Various iron and haem iron compounds were elevated in endometriosis	Involvement of HO-1 in regulating balance between iron-induced oxidative stress and endometriotic cyst fluid
Kizilay *et al*. (2017)	Animal: Albino Wistar rats	33	Blood, endo deposits		No difference in serum iron when injected with DFO, water or curcumin. DFO and curcumin affected cell proliferation	Curcumin with/without DFO reduced cell proliferation
Kobayashi *et al*. (2012)	Human	10	Eutopic endometrium, ectopic endometrium	Iron deposition, macrophage iron, oxidative stress, iron regulatory gene expression	Massive iron deposition in stroma of ovarian endometriosis, mostly in macrophages	Differential iron metabolism in ectopic endometrial stromal cells
Kokot *et al*. (2021)	Human		Serum	Oxidative stress markers (TAS, FRAP, albumin, bilirubin, uric acid, iron, SIRT, AOPP)	No difference in serum iron between EM/non-EM, lower serum iron in Stage IV EM compared to Stage III EM	
Li *et al*. (2020a)	Human	25	Follicular fluid	Transferrin, ferric irons	Reduced transferrin levels, increased follicular fluid ferric iron	Involvement of transferrin insufficiency and iron overload of follicular fluid in endometriosis-related infertility
Li *et al*. (2020b)	Human	32	Ectopic lesions	Ferroptosis, LPO, morphology, Ferroportin	Erastin can induce ferroptosis in ectopic endometrial stromal cells,	Role of FPN in treatment of endometriosis
Li *et al*. (2021b)	Human and mice	72	Peritoneal fluid	UIBC, Ferritin, Transferrin, TSAT, TIBC, ATP production, MMP, ROS, lipid peroxidation, RNA-seq	Iron overload disrupted blastocyst formation and induced lipid peroxidation via ferroptosis. Cytotoxicity was attenuated by ferroptosis inhibition. HMOX1 suppresses ferroptosis	HMOX1 suppresses ferroptosis and is upregulated in endometriosis providing a novel mechanism for treatment.
Li *et al*. (2022)	Human	48	Endometrioma, eutopic endometrium	VEGFA, IL8, HUVEC	Iron overload is associated with ferroptosis in endometriomas and upregulation of VEGFA, IL8, and HUVEC	Ferroptosis in endometriomas may trigger cytokine secretion and promote angiogenesis
Liang *et al*. (2022)	Human	39	Ectopic endometrial stromal cells	Cell viability, ferrous iron, lipid peroxidation, MDA,	MALAT1 was decreased during erastin-induced ferroptosis. MALAT 1 regulates MUC1-mediated ferroptosis suppression.	Targeting of the MALAT1/MUC1 pathway could be novel therapeutic strategy.
Liu *et al*. (2022)	Human	74	Serum, cyst fluid	Transferrin, iron, ferritin, UIBC	Iron and ferritin were higher in cyst fluid than in serum.	There is limited value in serum iron metabolites as a diagnostic biomarker of endometriosis.
Lousse *et al*. (2009)	Human	50	Peritoneal fluid	Macrophage ferritin, peritoneal iron, transferrin, ferritin, and prohepcidin	Iron storage in peritoneal macrophages is increased in endometriosis	Relevance to targeted therapies
Milewski *et al*. (2021)	Human	643	Peripheral blood	HMOX1 alleles	Endometriosis is associated with functional polymorphism of HMOX1	HMOX1 functional polymorphism may play a part in endometriosis pathogenesis
Mori *et al*. (2015)	Human	nr	Endometrium and ectopic lesions	Labile iron, catalytic iron, iron transport proteins	Higher catalytic iron in ectopic endometrial stromal cells than eutopic normal, lower ferroportin in eutopic ESCs	Ectopic ESCs play a protective role for cancer-target epithelial cells
Nagayasu *et al*. (2020)	Human	77	Cyst fluid		CF iron was higher in the group that was infertile due to endometriosis	Iron may play a role as a marker in predicting infertility in women with ovarian endometrioma
Ni *et al*. (2022)	Human and mice	nr	Murine granulosa cells, human follicular fluid	Ferroptosis markers within granulosa cells	Follicular fluid from women with endometriosis caused iron overload-induced ferroptosis *in vitro* and *in vivo*. Iron chelation alleviated endometriosis-related subfertility	Nuclear receptor coactivator four was involved in the ferroptosis mechanism and ferroptosis further suppressed oocyte maturity
Osman *et al*. (2012)	Human	38	Peritoneal fluid, serum	Iron levels	Serum iron lower in infertile endometriotic group serum but higher in peritoneal fluid	Role of oxidative stress in the development and progression of endometriosis and EM-related infertility
Polak *et al*. (2007)	Human	78	Peritoneal fluid	Lactoferrin levels	Lower peritoneal fluid lactoferrin in patients with minimal endometriosis than compared to controls and those with more severe endometriosis	Role of lactoferrin as a defence factor in peritoneal cavity
Polak *et al*. (2018)	Human	229	Peritoneal fluid	Hb, iron, total oxidative status, total antioxidant status	Hb, iron, and oxidative status higher in endometriosis peritoneal fluid. Total antioxidant values lower	Influence of impaired iron homeostasis on pathophysiology of peritoneal endometriosis
Rockfield *et al.* (2018)	Human	nr		NCO4, H-ferritin, p21, NCO4-B	Transformed endometriotic cells had higher migratory potential	Role of NCOA4 in transition into ovarian cancer
Sanchez *et al*. (2014)	Human	13	Follicular fluid	Total iron, L-ferritin, H-Ferritin, oocyte retrieval	Iron levels higher in endometriosis, ferritin expression varied depending on follicle location	Follicle aspiration at sites distant from endometrioma may increase probability of retrieving oocytes when surgical removal of endometrioma is not an option
Shigetomi *et al*. (2021)	Human	235	Cyst fluid	Total, haem and free iron, oxyhaemoglobin, methhaemoglobin, bilirubin	All iron types, methaemoglobin/oxyhaemoglobin ration and bilirubin, were higher in endometriosis.	Iron-induced oxidative stress may exceed the bilirubin-dependent antioxidant capability
Singh *et al*. (2013)	Human	340	Follicular fluid	ROS, NO, LPO, Iron, TAC,	Increased reactive oxygen species in patients who failed IVF	Possible benefits of multivitamin/mineral supplementation for patients undergoing IVF
Takahashi *et al*. (1996)	Human	24	Ovarian endometriomas	MRI cyst density, iron concentration	Density correlated with iron concentration	Role of MRI and T2 signal intensity in evaluating cyst fluid characteristics of endometriomas
Takenaka *et al*. (2017)	Human	9		Catalytic iron, O_2_ levels, IRP2	High iron deposition and IRP in ESCs and cysts. Increase in IRP2 expression upregulated intracellular iron.	Insufficient oxygen in cysts may cause stabilization of IRP2 against iron-mediated degradation
Thézénas *et al*. (2020)	Human		Ectopic lesions	Oxidative protein markers, LPO	All seen in ectopic lesions	AOC3 inhibitors had analgesic effects in inflammatory pain models, possible translational applicability
Van Langendonckt *et al*. (2002)	Human	70	Peritoneal fluid, serum samples, endometrium, endometriotic lesions, normal peritoneum	Iron levels, ferritin levels	Iron and ferritin higher in endometriotic PF. Levels varied based on peritoneum adjacent to differently coloured lesions	Relation of iron deposits to the presence of endometriotic lesions
Van Langendonckt *et al*. (2004)	Animal: Nude Mice	57	Endo lesions	Iron levels	No deposits found on glandular epithelium, low proliferative index in glandular epithelium	Iron conglomerates may trigger oxidative damage and chronic inflammation
Wan *et al*. (2022a)	Human	18	Endometrial stromal cells	Fibulin-1	Fibulin-1 showed increased expression in both eutopic and ectopic endometrium in women with endometriosis and promoted cell viability. Inhibition of fibulin 1 triggered ferroptosis-mediated cell death	The fibulin-1/ferroptosis pathway has an important role in endometriosis and may be a treatment target
Wan *et al*. (2022b)	Human and mice	17	Endometrial stromal cells	ADAMTS9-AS1 expression, MDA, ROS, GPX4	ADAMTS9-AS1 was upregulated in ectopic endometrium, and knockdown decreased cell viability. Ferroptosis inhibition blocked the effects of ADAMTS9-AS1	ADAMTS9-AS1 acts as a competing endogenous RNA and may be a therapeutic target
Wölfler *et al*. (2013)	Human	80	Peritoneal fluid	Haemopexin and haem	Haem levels not significantly different, no correlation between haem and haemopexin	Haemopexin downregulated in endometriotic PF.
Woo *et al*. (2020)	Human			Ferritin, MMP-2, ROS, NFkB	Overexpression of ferritin in endometriotic tissue	Contribution of iron to migration abilities of human endometriotic cells
Yamaguchi *et al*. (2008)	Human	36	Cyst fluid	Free iron, catalytic iron, LDH, lipid peroxidase, 8-OHdG	Increased free iron and iron deposits in endometriotic cysts	Abundant free iron possibly facilitation mutation rate and therefore malignant change
Yoshimoto *et al*. (2015)	Human	36	Endo cyst fluid	Total iron, haem iron, free iron	Higher iron related compound levels in endometriosis	Importance of iron-related compounds as biomarkers in malignant transformation of endometriosis
Zhou *et al*. (2022)	Human	103	Eutopic endometrium, ectopic endometrium	FAC, Ki67, ROS, PARP1 and SIRT1 expression	FAC inhibited cell growth, induced oxidative stress, and caused apoptosis. FAC impaired PARP1 expression.	Iron overload in ESCs may be involved in the inhibition of cell proliferation

nr, not reported; PF, peritoneal fluid; LDH, lactate dehydrogenase; 8-OHdG, 8-hydroxy-2′-deoxyguanosine; RANTES, regulated upon activation, normal T-cell expressed and presumably secreted; CRP, C-reactive protein; CA-125, cancer antigen 125; ROS, reactive oxygen species; NO, nitric oxide; LPO, lipid peroxidation; TAC, total antioxidant capacity; L-ferritin, light ferritin; H-ferritin, heavy ferritin; MDA, malondialdehyde; NF-kB, nuclear factor kappa-light-chain-enhancer of activated B cells; ESC, endometrial stromal cell; DMT1, divalent metal transporter-1; DFO, deferoxamine; IRP2, iron-responsive element-binding protein 2; O_2_, oxygen; Hb, haemoglobin; NCO4, nuclear receptor coactivator 4; PCV, packed cell volume; ESR, erythrocyte sedimentation rate; FPN, ferroportin; AOC3, amine oxidase, copper containing 3; CF, cyst fluid; MMP-2, matrix-metalloproteinase-2; TfR, transferrin receptor; OMA, ovarian endometrioma; oxyHb, oxyhaemoglobin; metHb, methaemoglobin; TAS, total antioxidant status; FRAP, ferric-reducing antioxidant power; EM, endometriosis; SIRT, sirtuin; AOPP, advanced oxidation protein products; UIBC, unsaturated iron-binding capacity; HMOX1, haem oxygenase-1; VEGFA, vascular endothelial growth factor A; IL8, interleukin-8; HUVEC, human umbilical vein endothelial cells; MALAT1, metastasis-associated lung adenocarcinoma 1; MUC1, mucin-1; ADAMTS9-AS1, ADAMTS9 antisense RNA 1; FAC, ferric ammonium citrate; Ki67, antigen KI-67; PARP1, poly [ADP-ribose] polymerase 1; TIBC, total iron-binding capacity.

### Bias and quality analysis

A formal methodological quality assessment was completed using the NOS. All studies were non-randomized and susceptible to selection bias. Just 18 of the 47 human studies accounted for the cycle phase in the reported methodology, and 31 described controlling for any other confounding variable such as age, comorbidity, or previous surgery, suggesting a high risk of confounding bias. A breakdown of the NOS scoring is presented in Table 2.

**Table 2.** Summary table of Newcastle–Ottawa scoring.

	Akashi *et al.*(2021)	Alizadeh *et al.* (2015)	Al-Shammaa (2020)	Alvarado-Díaz *et al.* (2015)	Alvarado-Díaz *et al.* (2016)	Arumugam (1994)	Arumugam and Yip (1995)	Atkins*et al.* (2018)	Bauckman *et al.* (2013)	Benaglia *et al.* (2015)	Chen *et al.* (2021)	Chmaj-Wierzchowska *et al.* (2013)	Defrère *et al.* (2006)	Fassbender *et al.* (2011)	Guo *et al.* (2015)	Hayashi *et al.* (2020)	Iizuka *et al.* (1998)	Imanaka *et al.* (2021a)	Imanaka *et al.* (2021b)	Imanaka *et al.* (2021c)	Kizilay *et al.* (2017)	Kobayashi *et al.* (2012)	Kokot *et al.* (2021)	Li *et al.* (2020a)	Li *et al.* (2020b)	Li *et al.* (2021b)	Li *et al.* (2022)	Liang *et al.* (2022)	Liu *et al.* (2022)	Lousse *et al.* (2009)	Milewski *et al.* (2021)	Mori *et al.* (2015)	Nagayasu *et al.* (2020)	Ni *et al.* (2022)	Osman *et al.* (2012)	Polak *et al.* (2007)	Polak *et al.* (2018)	Rockfield *et al.* (2018)	Sanchez *et al.* (2014)	Shigetomi *et al.* (2021)	Singh *et al.* (2013)	Takahashi *et al.* (1996)	Takenaka *et al.* (2017)	Thézénas *et al.* (2020)	Van Langendonckt *et al.* (2002)	Van Langendonckt *et al.* (2004)	Wan *et al.* (2022a)	Wan *et al.* (2022b)	Wölfler *et al.* (2013)	Woo *et al.* (2020)	Yamaguchi *et al.* (2008)	Zhou *et al.* (2022)	Yoshimoto *et al.* (2015)
**Is the case definition adequate?**	*	*	*	*	*	*	*	*	*	*	*	*	*	*	*	*	*	*	*	*	*	*	*	*	*	*	*	*	*	*	*	*	*	*	*	*	*	*	*	*	*	*	*	*	*	*	*	*	*	*	*	*	*
a) Yes, with independent validation*																																																					
b) Yes, e.g., record linkage or based on self-report																																																					
c) No description																																																					
**Representativeness of cases**	*	*	*		*		*	*		*	*		*		*	*		*	*	*	*		*	*	*	*	*	*	*	*	*		*	*	*	*	*		*		*	*			*	*	*	*	*			*	*
a) Consecutive or obviously representative series of cases*																																																					
b) Potential for selection bias or not stated																																																					
**Selection of controls**		*			*						*		*			*					*		*			*	*	*	*		*			*												*	*	*					*
a) Community controls*																																																					
b) Hospital controls																																																					
c) No description																																																					
**Definition of controls**		*	*		*		*	*	*		*		*	*		*						*	*	*		*	*	*	*	*	*	*		*			*				*				*	*	*	*		*		*	*
a) No history of disease (endpoint)*																																																					
b) No description of source																																																					
**Comparability of cases and controls on the basis of the design or analysis**																																																					
a) Study controls for phase cycle*					*		*						*	*		*					*			*	*	*				*					*	*	*		*						*	*		*					*
b) Study controls for any additional factor*		*				*		*		*	*	*	*	*		*			*		*		*	*	*	*	*	*	*	*	*			*	*		*	*	*	*	*				*	*		*					*
**Ascertainment of endometriosis**					*	*	*	*		*	*	*	*	*	*	*	*	*	*	*	*	*	*	*	*	*	*	*	*	*	*	*	*	*	*	*	*	*	*	*	*	*	*	*	*	*	*	*	*	*	*	*	*
a) Secure record (e.g. surgical or research record)*																																																					
b) Structured interview where blind to case/control status*																																																					
c) Interview not blinded to case/control status																																																					
d) Written self-report or medical record only																																																					
e) No description																																																					
**Same method of ascertainment for cases and controls**						*	*	*				*	*	*	*	*	*	*	*		*	*	*	*	*	*	*	*		*	*	*	*	*	*	*	*			*	*				*	*	*	*		*		*	*
a) Yes*																																																					
b) No																																																					
**Non-response rate**	*	*	*	*		*	*	*	*	*	*	*	*	*	*	*	*	*	*	*	*	*	*	*	*	*	*	*	*	*	*	*	*	*	*	*	*	*	*	*	*	*	*	*	*	*	*	*	*	*	*	*	*
a) Same rate for both groups*																																																					
b) Non-respondent rate described																																																					
c) Rate different between cases and controls with no description																																																					
**Total score**	3	6	4	2	6	5	7	7	3	5	7	5	9	7	5	9	4	5	6	4	8	5	8	8	7	9	8	8	7	8	8	5	5	8	7	6	8	4	6	5	7	4	3	3	8	9	7	9	4	5	3	6	9

Each asterisk (*) denotes satisfaction of the corresponding criterion and provides one point to the overall quality score.

### Systemic iron levels

Seven studies reported on systemic iron levels (Osman *et al.*, 2012; Chmaj-Wierzchowska *et al.*, 2013; Alizadeh *et al.*, 2015; Al-Shammaa, 2020; Kokot *et al.*, 2021; Liu *et al.*, 2022). Five studies compared serum iron levels in women with and without endometriosis, and one used an animal model of endometriosis (Atkins *et al.*, 2018).

Two small case–control studies with significant methodological weaknesses (Table 2) reported higher serum iron levels in women with endometriosis (Alizadeh *et al.*, 2015; Al-Shammaa, 2020) while, in contrast, another study reported lower serum iron levels (Osman *et al.*, 2012). Iron deficiency and secondary anaemia were demonstrated in Macaques with naturally occurring endometriosis (Atkins *et al.*, 2018), where duodenal, bone marrow, and liver sampling supported a systemic deficiency and correction was attempted through increased gastrointestinal absorption, as evidenced by ferroportin-1 upregulation despite high dietary iron.

The remaining three studies found no significant difference in serum iron levels between women with endometriosis and controls (Chmaj-Wierzchowska *et al.*, 2013; Kokot *et al.*, 2021; Liu *et al.*, 2022). Of particular note, however, the only study that considered disease severity did demonstrate serum iron deficiency in women with revised American Fertility Society (rAFS) grade IV endometriosis (Kokot *et al.*, 2021). Finally, one study (Liu *et al.*, 2022) included a comparison of iron levels in serum and ovarian endometriomas. The iron excess found in endometriomas was not observed in the serum, suggesting that the iron overload is limited to the locality of endometriotic tissues. Overall, the included studies' findings were contradictory and marred by low quality. Specifically, none characterized the patient population by menstrual cycle phase or for hormonal treatments. At most, there is possible evidence of an association between increased disease severity and systemic iron deficiency.

### Iron in peritoneal fluid

Despite using different methodologies and patient characteristics, six studies found evidence of iron overload in the peritoneal fluid of endometriosis patients, compared to healthy controls (Arumugam and Yip 1995; Van Langendonckt *et al.*, 2002; Lousse *et al.*, 2009; Osman *et al.*, 2012; Polak *et al.*, 2007, 2018).

Free iron and ferritin levels were significantly higher in peritoneal fluid of patients with endometriosis compared with that of healthy controls (Arumugam and Yip 1995; Van Langendonckt *et al.*, 2002; Lousse *et al.*, 2009). Furthermore, a local rather than systemic source was suggested for the observed peritoneal iron overload, as evidenced by comparatively low serum iron levels (Van Langendonckt *et al.*, 2002; Osman *et al.*, 2012).

Increasing disease severity significantly correlated with iron excess (Stage III–IV vs Stage I–II; rAFS classification) in some studies (Arumugam and Yip 1995; Polak *et al.*, 2007, 2018), while others found no significant difference (Lousse *et al.*, 2009). The high iron and ferritin levels were reported to be specific to the secretory phase by some studies (Van Langendonckt *et al.*, 2002), while others did not detect such a difference in any marker of iron metabolism (Lousse *et al.*, 2009; Polak *et al.*, 2007, 2018).

While all studies reported iron overload in the peritoneal fluid of women with endometriosis, there is no consensus on the effect of the menstrual cycle stage or disease severity on iron concentrations. Moreover, multiple studies suggested that the excess iron is produced locally rather than systemically.

### Iron in the peritoneum and peritoneal deposits

The available data on iron in the peritoneum and peritoneal deposits in endometriosis is limited, with only three studies reporting iron levels in these tissues. Two studies examined the peritoneum of women (Van Langendonckt *et al.*, 2002; Fassbender *et al.*, 2011), while one used a nude mice model (Defrère *et al.*, 2006).

Higher iron and ferritin levels were reported in the peritoneum adjacent to established endometriotic lesions (Van Langendonckt *et al.*, 2002). When lesions were divided into newer and older lesions, as defined by their visual appearances, all demonstrated raised iron levels, suggesting persistent but minimally variable iron excess throughout the natural history of peritoneal disease. ‘Typical features’ of iron excess were also seen in peritoneal lesions and adjacent tissues in a mouse model of endometriosis (Defrère *et al.*, 2006). Furthermore, the authors suggested iron overload, secondary to the lysis of erythrocytes likely by local macrophages, due to the comparably high concentration of siderophages (haemosiderin-laden macrophages). A reduced expression of ferritin mRNA in macroscopically normal peritoneum was detected in women with endometriosis, suggesting that the iron overload is limited to peritoneal lesions and does not extend into surrounding tissues (Fassbender *et al.*, 2011). Overall, all studies support the presence of localized iron overload in peritoneal endometriotic lesions.

### Iron content in ovarian endometriomas

The iron content of ovarian endometriomas is well studied, with 11 papers reporting on the iron concentrations in this tissue (Takahashi *et al.*, 1996; Iizuka *et al.*, 1998; Yamaguchi *et al.*, 2008; Singh *et al.*, 2013; Sanchez *et al.*, 2014; Benaglia *et al.*, 2015; Guo *et al.*, 2015; Yoshimoto *et al.*, 2015; Nagayasu *et al.*, 2020; Imanaka *et al.*, 2021a,b). The findings of Benaglia *et al.* (2015), Sanchez *et al.* (2014), Nagayasu *et al.* (2020), and Singh *et al.* (2013) are summarized elsewhere in this review.

While some studies have compared iron levels in endometriomas to other benign ovarian cysts, others have compared them with malignant ovarian lesions. Endometriomas had significantly higher levels of total, haem, and free iron when compared with serous/mucinous adenomas and mature teratomas (Iizuka *et al.*, 1998, Imanaka *et al*., 2021b). They also have higher iron levels (total, haem, and free) compared with clear cell ovarian cancers, and serous/mucinous adenomas (Yamaguchi *et al.*, 2008) and with a pooled group of endometriosis-associated ovarian cancers (EAOCs) (Yoshimoto *et al.*, 2015). Alternatively, comparably high iron levels were found in endometrioid ovarian adenocarcinomas, haemorrhagic corpus luteum, and lutein cysts (Iizuka *et al.*, 1998).

Taking a temporal approach, when ‘older’ and ‘younger’ endometriomas were compared based on their visual appearance during surgery, a significantly higher level of free iron and ferritin was observed in ‘older’ cysts Guo *et al.* (2015). The accuracy of this categorization, however, remains to be verified, since the appearance may be a mere reflection of hormone responsiveness or aberrant angiogenesis of the lesions. Two studies investigating specific iron-sensitive MRI techniques as a diagnostic tool for endometriomas (Takahashi et al., 1996; Imanaka *et al.*, 2021b) also confirmed higher iron levels in endometriomas via cyst fluid sampling.

Overall, all studies on endometriomas have reported elevated levels of iron and iron-related proteins in endometriotic fluid compared to almost all other ovarian cyst subtypes. The only exception was alternative haemorrhage-associated cysts, which suggests endometriotic bleeding and haem catabolism to be the causative pathway for the subsequent iron excess. Furthermore, the reported temporal association with older, more established endometriomas and higher iron levels suggest accumulation due to failed iron sequestration mechanisms over time. Since the origin of iron in endometriomas is therefore localized bleeding at the time of menstruation, it appears to be related to the presumed cyclical hormone responsiveness in this sub-type of endometriosis.

### Ovarian follicle iron content

Four studies reported iron levels within ovarian follicles (Singh *et al.*, 2013; Sanchez *et al.*, 2014; Benaglia *et al.*, 2015; Li *et al.*, 2020a). All studies included a subfertile population undergoing IVF and examined follicular fluid sampled at the stage of oocyte retrieval.

Significantly higher levels of follicular free iron (Singh *et al.*, 2013) and ferric iron in addition to lower transferrin levels with transferrin saturation all indicated ‘iron overload’ (Li *et al.*, 2020a) in women with endometriosis compared to those with tubal infertility. These findings suggest that high local iron levels may lead to transferrin saturation with subsequent insufficiency in endometrioma-adjacent follicles.

In women with unilateral endometriomas, higher levels of free iron and ferritin were observed in affected ovaries compared to healthy ones (Benaglia *et al.*, 2015) and a stepwise increase has been reported in iron levels within the normal ovaries through to spatially distant follicles in the diseased ovaries and, finally, endometrioma-adjacent follicles (Sanchez *et al.*, 2014).

Overall, these four studies confirm localized iron overload in and adjacent to endometriotic lesions, which may contribute to subfertility in women with endometriosis.

### Iron and macrophages

Macrophage iron concentration has been examined in three studies (Lousse *et al.*, 2009; Kobayashi *et al.*, 2012; Akashi *et al.*, 2021). The observation of a higher ferritin levels in peritoneal macrophages, particularly in the secretory phase in women with endometriosis, has been interpreted as a progressive overwhelming of the iron-detoxification mechanisms during the menstrual cycle (Lousse *et al.*, 2009). Eutopic endometrial stroma of women with endometriosis also had a high deposition of iron-laden macrophages (Kobayashi *et al.*, 2012).

Iron-laden macrophages were also found in the epithelial layers of ovarian endometriomas and ovarian clear-cell carcinomas, which concomitantly but predictably expressed significantly raised Ki-67 levels (Akashi *et al.*, 2021).

### Iron regulation and dysregulation

Iron levels reach excess when the mechanisms controlling iron homeostasis fail or are overwhelmed. Five studies examined alterations in iron transport and inflammatory pathways in endometriotic tissues (Kobayashi *et al.*, 2012; Alvarado-Díaz *et al.*, 2015, 2016; Takenaka *et al.*, 2017; Akashi *et al.*, 2021).

Iron regulatory genes have demonstrated alterations in ectopic endometriotic cell lines (Kobayashi *et al.*, 2012), where divalent metal transporter 1 (*DMT1*), F-box and leucine-rich repeat protein 5 (*FBXL-5*), cullin 1 (*CUL1*), hypoxia-inducible factor 1 beta (*HIF1B*), iron regulatory proteins 1 and 2 (*IRP1*, *IRP2*), and ferroportin (*FPN*) were upregulated while hypoxia-induced factor 2A (*HIF2A*) was downregulated.

Iron overload induced greater expression of two subtypes of DMT1, which is responsible for iron influx into cells (Alvarado-Díaz *et al.*, 2016). An increase in DMT1 protein expression was observed in ovarian endometriomas and clear-cell adenocarcinomas (Akashi *et al.*, 2021). However, the levels of proteins encoded by the genes *DMT-1*, *FPN*, and *IRP1* showed no difference between endometriomas and normal endometrium (Takenaka *et al.*, 2017). *IRP2* was the only gene to show consistent upregulation. IRP2 plays a key role in cellular iron homeostasis by altering transferrin levels dependent on intracellular iron levels (Zhang *et al.*, 2014). In cell lines with proven iron excess, IRP2 expression decreased, as would be expected. However, in a hypoxic environment, IRP2 remained unaltered, suggesting that in endometriosis, the altered iron metabolism and failure of the normal homeostatic pathways may directly result from tissue hypoxia.

Furthermore, when isolated endometrial stromal cells from healthy women are exposed to iron excess, stimulation of the pro-inflammatory NF-κB pathway is evidenced (Alvarado-Díaz *et al.*, 2015). Taken together, the studies suggest aberrant iron regulation and transport in endometriotic tissues, with increased iron import and decreased iron export.

### Oxidative–antioxidative balance in endometriosis

Iron-mediated oxidative stress (IMOS) occurs due to the formation of toxic hydroxyl radicals in environments of iron excess and this was explored in 13 studies (Arumugam and Yip, 1995; Yamaguchi *et al.*, 2008; Singh *et al.*, 2013; Alizadeh *et al.*, 2015; Polak *et al.*, 2018; Al-Shammaa, 2020; Hayashi *et al.*, 2020; Thézénas *et al.*, 2020; Woo *et al.*, 2020; Kokot *et al.*, 2021; Shigetomi *et al.*, 2021; Milewski *et al.*, 2021; Zhou *et al.*, 2022).

#### Systemic studies

Three studies examined systemic markers of oxidative stress (Alizadeh *et al.*, 2015; Al-Shammaa, 2020; Kokot *et al.*, 2021). Some studies reported no significant differences in the oxidative stress markers such as malondialdehyde (MDA) and carbonyl (Alizadeh *et al.*, 2015) between patients and controls, while others reported significantly higher serum levels of MDA and 8-hydroxy-2-deoxy guanosine (8-HdG) in the disease cohort (Al-Shammaa, 2020). Some other non-endometriosis specific systemic antioxidants such as ferric-reducing antioxidant power, advanced oxidation protein products, and telomerase levels were also reported to be higher in endometriosis patients compared to controls, but these were also raised in other benign inflammatory gynaecological pathologies (Kokot *et al.*, 2021). Multiple other antioxidant markers were reported to be unchanged. Therefore, the limited existing evidence related to systemic oxidative stress provides no consensus.

#### Studies examining local oxidative stress

The data related to localized oxidative stress in endometriosis are robust, with studies examining IMOS in bio-samples local to endometriotic lesions, including peritoneal fluid and endometriotic deposits. These studies report on multiple markers of oxidative stress, including MDA, 8-HdG, 4-hydroxynonenal (4-HNE), lactate dehydrogenase (LDH), lipid peroxidation (LPO), total oxidative status (TOS), reactive oxygen species (ROS), and nitric oxide (NO), as well as antioxidants such as total antioxidant capacity (TAC), superoxide dismutase (SOD), catalase, glutathione peroxidase (GPx), and glutathione reductase (GR).

MDA levels in women with mild or severe endometriosis and controls were similar in one study (Arumugam and Yip, 1995); yet, another reported a significantly higher TOS in Stage I, III, and IV endometriosis patients compared to controls and a significant correlation between TOS and iron levels (Polak *et al.*, 2018). Conversely, the antioxidant marker TAS was significantly lower in endometriosis patients, but this finding was limited to patients with Stage IV disease.

Oxidative stress markers, including LDH, LPO, and 8-HdG, were significantly higher in endometriotic cysts and positively correlated with higher free iron levels (Yamaguchi *et al.*, 2008). Iron overload in endometriotic stromal cells was associated with oxidative stress but iron excess appeared to inhibit cell proliferation and increase autophagy of endometriotic cells (Zhou *et al.*, 2022). IMOS has been shown to exceed the ability of a bilirubin-dependent antioxidant pathway to maintain the oxidative-antioxidative balance in endometriotic tissue (Shigetomi *et al.*, 2021).

In the context of endometriosis-related infertility, markers of oxidative stress, such as ROS, NO, and MDA, were significantly raised (Singh *et al.*, 2013), while antioxidant markers TAC, SOD, catalase, GPx, and GR were all significantly lower. Haem oxygenase 1 (HMOX-1), an enzyme responsible for the catabolism of haemoglobin and known to be protective against inflammation and oxidative stress, was also found to have a functional polymorphism in women with endometriosis (Milewski *et al.*, 2021). In a murine model of endometriosis, increased levels of 8-HdG and 4-HNE (a more IMOS-specific marker) were associated with lower FSH levels and the number of viable foetuses, suggesting a link with endometriosis-related subfertility (Hayashi *et al.*, 2020).

Overall, the available studies suggest that oxidative stress is prevalent in endometriosis and there is consensus evidence of deviation in the oxidative–antioxidative balance. While excess iron in the lesions appears to be associated with this alteration, direct causation of oxidative stress is hard to prove, and non-iron-mediated pathways may also be involved.

### Ferroptosis

Ferroptosis, defined as a distinct form of regulated cell death via iron-dependent lipid peroxidation (Jiang *et al.*, 2021), represents a recent area of interest in endometriosis pathophysiology. The overproduction of iron-induced reactive oxygen species is the defining event in ferroptosis and is the cause of this recently identified mode of cell death. This review includes eight studies published in the last three years which report on the role of ferroptosis in endometriosis (Li *et al.*, 2020b, 2021a,b, 2022; Liang *et al.*, 2022; Ni *et al.*, 2022; Wan *et al.*, 2022a,b).

#### Ferroptosis in endometriosis pathogenesis

Erastin, an established inducer of ferroptosis (Zhao *et al.* 2020), was found to increase the rate of ferroptosis in ectopic endometriotic stromal cells but not in normal eutopic endometrial stroma, suggesting that pathophysiological limited resistance to ferroptosis may be the pathway allowing the establishment of ectopic endometrium (Li *et al.*, 2020b).

Several studies have investigated the mechanisms underlying resistance to ferroptosis in endometriotic stromal cells. Downregulation of the gene *MALAT1* (Cai *et al.*, 2020) in erastin-induced ferroptosis in these cells (Liang *et al.*, 2022) suggests that ferroptosis is suppressed by a MALAT1-mediated mechanism. Overexpression of the long non-coding *RNA ADAMTS9-AS1* in ectopic endometrial tissue was associated with enhanced cell viability via a reduction in ferroptosis (Wan *et al.*, 2022a). Inhibiting ferroptosis with ferrostatin-1 reversed the ADAMTS9-AS1-mediated cell survival in stromal cells, suggesting a potential treatment target (Wan *et al.*, 2022a).

Interestingly, ferroptosis may unexpectedly lead to inflammation and neovascularisation in endometriotic stromal cells. Induction of ferroptosis in endometriotic stromal cells upregulated the expression of pro-inflammatory and angiogenic cytokines, such as IL-8 and vascular endothelial growth factor A (VEGFA), suggesting that ferroptosis may support the establishment and growth of endometriotic lesions (Li *et al.*, 2022).

Finally, fibulin-1, a glycoprotein involved in extracellular matrix stabilization, may play a role in the resistance to ferroptosis in endometriotic stromal cells (Forti *et al.*, 2002; Timpl *et al.*, 2003; Liu *et al.*, 2016; Holmila *et al.*, 2017), since overexpression of fibulin-1 in endometriotic stromal cells inhibited ferroptosis. Conversely, inhibition of fibulin-1 increased ferroptosis within endometriotic stromal cells (Wan *et al.*, 2022b), suggesting a potential therapeutic strategy for endometriosis. These studies propose several mechanisms for altered regulation of ferroptosis in patients with endometriosis and suggest that aberrant resistance to ferroptosis is a critical factor allowing ectopic endometrial establishment and growth. They also suggest that ferroptosis is involved in cell proliferation, survival, and angiogenesis, thereby contributing to the establishment of ectopic endometriotic lesions.

#### Ferroptosis in endometriosis-associated subfertility

Two studies in murine models explored the role of ferroptosis in endometriosis-associated subfertility (Li *et al.*, 2021b, Ni *et al.*, 2022). When mouse embryos were exposed to the peritoneal fluid of women with endometriosis, mouse fertility was reduced, ostensibly, due to increased levels of ferroptosis (Li *et al.*, 2021b). Ferrostatin-1, a ferroptosis inhibitor (Cao and Dixon, 2016; Miotto *et al.*, 2020), and HMOX1 (Li *et al.*, 2021b) may have a possible protective role in reversing the effect on fertility. Similarly, murine oocytes exposed to peritoneal fluid from endometriosis women showed iron overload-induced ferroptosis *in vitro* and *in vivo*, and exosomes released from granulosa cells affected by ferroptosis, further suppressed the maturation of oocytes (Ni *et al.*, 2022). This limited data suggest that ferroptosis is involved in initiating inflammation and affects oocytes and blastocysts, thus promoting the common symptoms associated with the disease.

### Iron and symptoms

Total, haem, and free iron levels in endometriomas were found to correlate with the severity of dysmenorrhoea (Imanaka *et al*., 2021a). Total and haem median iron concentrations in endometrioma content were significantly associated with symptom severity, while a similar but non-significant trend was observed for free iron concentrations, suggesting that iron may play an important role in the pro-inflammatory pain pathways in endometriosis.

### Iron and infertility

Eight studies examined the association between iron levels and infertility in women with endometriosis (Arumugam, 1994; Singh *et al.*, 2013; Sanchez *et al.*, 2014; Benaglia *et al.*, 2015; Hayashi *et al.*, 2020; Li *et al.*, 2020a; Nagayasu *et al.*, 2020; Chen *et al.*, 2021).

A novel murine model replicating ovarian endometriosis was found to have significantly higher levels of iron within the ovarian tissue and these mice were less fertile than controls (Hayashi *et al.*, 2020), proposing that oxidative stress from iron excess directly and negatively impacts folliculogenesis, reducing fertility. These findings are, however, discordant with the human studies, which found no significant differences in oocyte quality or retrieval rate between women with high and low iron levels (Sanchez *et al.*, 2014; Benaglia *et al.*, 2015).

Significantly higher levels of follicular fluid iron in women with endometriosis undergoing IVF were reported when compared with women with tubal infertility (Singh *et al.*, 2013). Follicular fluid from women with endometriosis caused a significantly lower *in vitro* oocyte maturation rate compared to fluid from controls, and the addition of transferrin to bind excess iron proved reversibility, demonstrated by an improved maturation rate (Li *et al.*, 2020a).

Iron exposure significantly impaired murine embryo development *in vitro*, with rates of both apoptosis and ferroptosis positively associated with iron concentration (Chen *et al.*, 2021). Women with endometriosis-associated infertility had significantly higher levels of iron within their endometriomas (Nagayasu *et al.*, 2020) than women with no signs of infertility, suggesting a role of iron in endometriosis-associated infertility. The infertile group in this study was significantly younger, and thus, this observational study may have demonstrated age-related iron levels in endometriomas as opposed to a true association with infertility.

When the effect of iron on male fertility was investigated by exposing healthy spermatozoa from men with proven fertility to the peritoneal fluid extracted from women with and without endometriosis, significantly lower rates of successful acrosome reactions alongside significantly higher concentrations of free iron in the peritoneal fluid were observed in the endometriosis group (Arumugam, 1994). These findings were limited to Stage III and IV endometriosis.

Although there is some evidence to suggest that iron excess may play a role in reducing fertility, overall, the studies investigating the relationship between iron levels and infertility in endometriosis have produced mixed results.

### Iron chelation

Given the key role of iron in the pathogenesis of endometriosis, it is an attractive target for potential therapeutics. Four animal studies report the action of iron chelators in endometriosis (Defrère *et al.*, 2006; Kizilay *et al.*, 2017; Chen *et al.*, 2021; Ni *et al.*, 2022). Iron chelators, like deferoxamine (DFO), bind ferric iron, forming stable inactive complexes. Injections of DFO in a murine endometriosis model found no change in the total number of endometriotic lesions but demonstrated reduced levels of iron in those lesions and a decreased proliferative index (Ki-67 immunostaining) (Defrère *et al.*, 2006). Furthermore, intra-peritoneally injected DFO and curcumin demonstrated implant size to significantly decrease with curcumin alone or with a combination of DFO and curcumin in a mouse model (Kizilay *et al.*, 2017). Curcumin is the active molecule within the turmeric plant, which has established antioxidant and iron-binding properties.

When DFO and vitamin E were used in conjunction, iron-mediated oocyte dysmaturity was ameliorated in mice via a reduction in ferroptosis (Ni *et al.*, 2022). Similarly, iron chelation also partially reversed murine blastocyst dysfunction suggesting that excess peritoneal iron is likely to play a role in endometriosis-associated infertility (Chen *et al.*, 2021).

## Discussion

**Figure 3.**
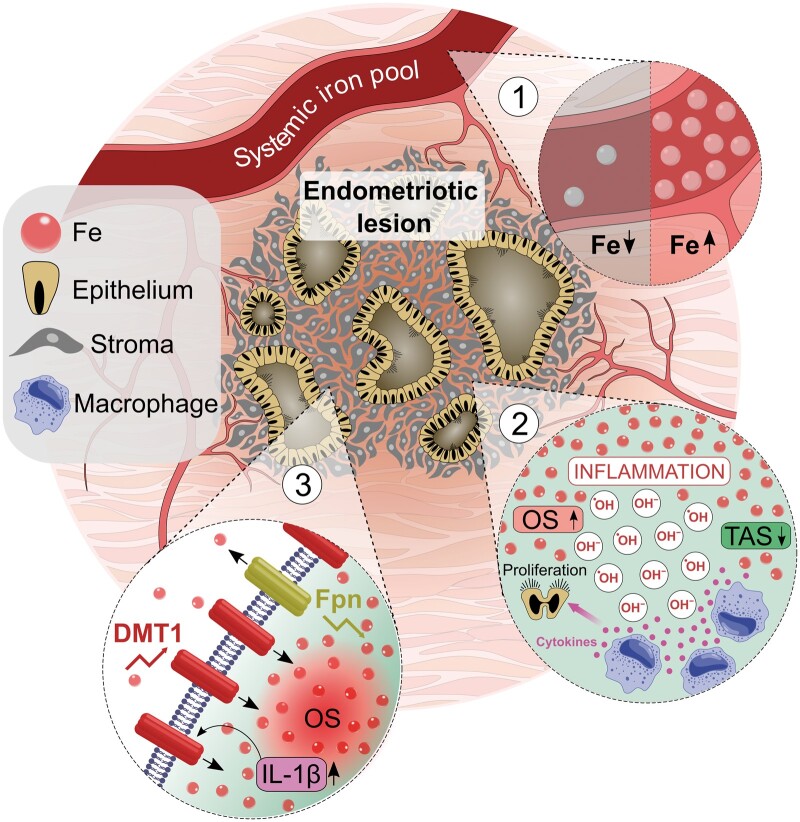
**Pathophysiology of iron in endometriosis.** Schematic diagram highlighting the most established pathways involved in aberrant iron physiology in endometriotic tissues. (1) Conflicting evidence regarding systemic iron levels. (2) Oxidative stress and inflammation. (3) Abnormal iron transport. Fe, iron; OS, oxidative stress; TAS, total antioxidant status; Fpn, ferroportin; DMT1, divalent metal transporter 1; IL-1β, interleukin 1 beta.

This review presents a summation of the current evidence regarding the role of iron in the pathophysiology of endometriosis. Localized iron excess appears to be an established feature of all ectopic endometriosis lesions. Within these lesions, oxidative stress is strongly associated with elevated iron levels, and aberrant expression of iron-transport proteins appears to be one mechanism responsible for maintaining iron excess. Iron-mediated oxidative stress is implicated in the development of a pro-inflammatory micro-environment, which is linked to subfertility, symptom severity, and, possibly, malignant transformation. The role of iron in the systemic circulation is less clear, with limited studies suggesting conflicting results. Figure 3 presents the pathophysiological mechanisms highlighted by this review.

The overarching viewpoint afforded through this systematic review enables a greater appreciation of the interplay between pathways and mechanisms relevant to iron, which may facilitate endometriosis establishment and progression, thus, allowing the postulation of novel theories for the pathogenesis and identification of potential therapeutic strategies.

Pathophysiological changes at the peritoneal-endometriosis interface are posited to play an important role in allowing endometriosis deposits to develop and iron appears to play a significant role in this process. We propose that the presence of retrograde menstruation and subsequent hormonally influenced recurrent bleeding from endometriotic tissue leads to iron excess via erythrocyte degradation. Consequential oxidative stress produces a pro-inflammatory state, associated with an abnormal resistance to ferroptosis, which encourages homeostatic dysregulation and hypoxia resistance, inciting endometriotic tissue to proliferate at an ectopic site. This review provides evidence for the existence of each step in this pathway.

Iron overload is amply demonstrated in ovarian endometriomas, peritoneal endometriosis, and the peritoneal fluid of women with endometriosis. Elevated levels of erythrocytes and haemoglobin are found in the peritoneal fluid of endometriosis patients and have previously been ascribed to either haemorrhage from ectopic lesions or aberrant processing of menstrual effluent during retrograde menstruation (Halme *et al.*, 1984; D'Hooghe and Debrock, 2002; Van Langendonckt *et al.*, 2004; Polak *et al.*, 2018). Bleeding, as a source of iron, is supported by the findings in in this review, whereby all ovarian lesions associated with haemorrhage, including endometriosis, endometrioid-type adenocarcinomas, and a haemorrhagic corpus luteum, demonstrated an iron-rich micro-environment.

The above is logical, considering that senescent erythrocytes release iron during erythrophagocytosis where they are engulfed by peritoneal macrophages and undergo degradation and recycling (Gupta *et al.*, 2015). Haem is catabolized via interaction with HMOX-1 to release free iron, which within normal physiological conditions is rapidly stored within the stable ferritin complex or transported extracellularly via transferrin for further processing (Ganz and Gordon, 2016). However, given the excessive levels of free and stable iron complexes demonstrated in the included studies, we can conclude that these homeostatic processes are either overwhelmed or defective in endometriosis.

Altered iron transport may also have a role in the maintenance of iron excess. As outlined, cellular iron importers such as DMT1 appear upregulated in endometriosis, while the iron exporter ferroportin is downregulated. Increased IL-1β levels are also associated with the upregulation of DMT1, leading to a pathological circular pathway of DMT1 upregulation leading to cellular iron influx and induction of oxidative stress (Alvarado-Díaz *et al.*, 2016). This, in turn, leads to IL-1β-mediated inflammation and over-expression of DMT1. Coupled with the finding of ferroportin downregulation in endometriosis (Li *et al.*, 2021b), abnormal iron transport does appear to play a role in iron excess. The cause of altered iron-transport regulation is unclear but may suggest a genetic predisposition to endometriosis. However, the studies are limited, and the suggested mechanisms remain primarily theoretical. Alternative explanations for these findings have not been investigated and may be a fruitful avenue for further research.

### Oxidative stress

Iron disrupts redox homeostasis and leads to the formation of hydroxyl radicals. Hydroxyl molecules are highly toxic and, on formation, oxidize any nearby chemical group capable of reaction, including DNA, lipids, and proteins, leading to cell death or DNA mutations (Galaris *et al.*, 2019). Cellular and tissue damage is the result, and oxidative stress is implicated in malignancies, atherosclerosis, and chronic inflammation (Pizzino *et al.*, 2017). Within endometriosis, oxidation has been linked with infertility, inflammation, and malignant transition (Gupta *et al.*, 2006; Scutiero *et al.*, 2017).

The included studies are primarily in accord with one another in describing high levels of oxidative stress in and around endometriotic lesions. The findings of equivalent TOS but deficient TAS in endometriosis suggest a deficiency in the defence against oxidation rather than an overwhelming formation of oxidative radicals (Polak *et al.*, 2018). Furthermore, the progressive and cumulative deterioration in the oxidative balance demonstrated with disease severity, and the volume of ectopic tissue within the peritoneum is in keeping with the cumulative snowballing effect of initial lesion establishment to facilitate the disease progression reported in primate studies (Fazleabas *et al.*, 2002; Hapangama *et al.*, 2010).

The theory that the presence of oxidative stress alone may permit the maintenance and proliferation of endometriotic lesions (Pirdel and Pirdel, 2014) has been supported by a murine model (Defrère *et al.*, 2006) where iron levels were not associated with the establishment of lesions but iron excess supported their maintenance and proliferation. Macrophages produce pro-inflammatory cytokines in response to haem and iron (Simoni *et al.*, 1994) and stimulate carbon monoxide (CO) production. CO is a potent vasodilator and has been theorized to stimulate angiogenesis in endometriosis (Polak *et al.*, 2018), thereby creating a hospitable environment for the development of lesions. This may partly explain why some lesions proliferate and thrive whilst others do not. The relationship between iron excess and oxidative stress is well described in the included papers and the broader literature (Donnez *et al.*, 2016; Galaris *et al.*, 2019; Hayashi *et al.*, 2020; Ng *et al.*, 2020). The evidence, however, seems to be incongruous, suggesting that iron excess and oxidative stress could play a causative role as well as being a consequence of endometriosis. We postulate that menstrual effluent after retrograde menstruation will initiate an iron excess and oxidative stress at the initial ectopic sites, facilitating the establishment of new lesions, while the established lesions with an iron over-load (even between menses) maintain oxidative stress and, thus, cause lesion progression and contribute to symptoms. Oxidative stress and ferroptosis represent the two major pathways through which iron excess may feed into pro-inflammatory and apoptotic-resistant pathways and are worth exploring in greater detail. Combination therapy to reduce iron overload and anti-inflammatory medications is likely to produce cumulative benefit for the patients and this is an important avenue of future research.

Beyond the pro-inflammatory effects of oxidative stress are the potential genetic mutations noted in malignancies (Hayes *et al.*, 2020). Oxidative stress has been suggested as a direct cause of malignant transformation (Yamaguchi *et al.*, 2008) and EAOCs, such as endometrioid and clear cell ovarian malignancies, have been linked to oxidative stress (Dahiya *et al.*, 2021). DNA damage from IMOS has been proposed as the likely causative factor (Iwabuchi *et al.*, 2015; Taniguchi, 2017). The pathway from iron excess to oxidative stress is, therefore, a potential preventative target for malignant transformation. Although highly proliferative ovarian cancers contained iron laden macrophages, EAOCs generally seem to have lower iron levels than endometriomas and the malignant transformation of endometriosis is a relatively rare event. Until a comprehensive cellular transcriptomic, metabolomic, proteomic, and mutational signature of ectopic endometriotic cells in different endometriosis sub-types are directly compared against the sub-regions of the eutopic endometrium, it is difficult to draw conclusions about the exact and specific cellular differences in endometriosis lesions. Therefore, with the current evidence, it is difficult to decide whether chelation of iron could reduce the risk of the relatively rare, malignant transformation of endometriomas, and further research is required to clarify this possibility.

Oxidative stress has also been implicated in endometriosis-associated subfertility by several high-quality studies (Singh *et al.*, 2013; Hayashi *et al.*, 2020; Li *et al.*, 2020a). Ovarian follicles with iron-rich environments demonstrated lower-quality immature embryos, and animal models confirmed fewer viable foetuses (Hayashi *et al.*, 2020; Li *et al.*, 2020a; Nagayasu *et al.*, 2020). Furthermore, there was evidence of reversibility, since oocyte maturation rates were significantly improved with the introduction of transferrin to bind and stabilize free iron (Li *et al.*, 2020a). IMOS is, therefore, a significant factor in endometriosis-associated subfertility and a highly attractive pathway to target in future research.

### Ferroptosis

Iron-mediated cell death was recognized as a novel mechanism as late as 2012 (Dixon *et al.*, 2012), and as such, this is an evolving area of research. Iron overload is the primary driver for this form of regulated cell death, and endometriotic lesions establish and thrive in an iron-rich environment. This review highlights the original work studying the role of ferroptosis in endometriosis and the potential mechanisms leading to possible resistance.

The available data suggests aberrant resistance to ferroptosis in endometriotic tissues. The abnormal regulation or resistance to ferroptosis in endometriosis has been suggested (Ng *et al.*, 2020) and a link between hypercholesterolaemia and ferroptosis has been posited. Since cholesterol-derived lipophilic antioxidants are a source of protection from ferroptosis (Shimada *et al.*, 2016), elevated cholesterol levels in the peritoneal fluid of women with endometriosis (Sharma *et al.*, 2010) have been proposed to be a potential mechanism of abnormal ferroptosis resistance (Ng *et al.*, 2020). This theory was supported by studies, demonstrating 3-hydroxy-3-methylglutaryl coenzyme A reductase inhibitors, or statins, in the treatment of endometriosis (Villanueva *et al.*, 2013; Taylor *et al.*, 2017; Sokalska *et al.*, 2019).

The ferroptosis pathway holds promise as a potential treatment target. Inhibition of ferroptosis with ferrostatin-1 was associated with improved fertility outcomes in mice (Li *et al.*, 2021b), but resistance to ferroptosis appears to be associated with increased viability of endometriotic cells (Wan *et al.*, 2022a). Therefore, the relationship between ferroptosis resistance and the clinically apparent symptoms remains poorly delineated and requires further research.

### Hypoxia resistance

The uterus is a highly vascular organ and eutopic endometrial physiology is finely regulated by changes in oxygen concentration (Maybin *et al.*, 2018). Eutopic endometrial cells have high oxygen levels for normal physiological function (Reavey *et al.*, 2021), and thus, unsurprisingly, hypoxia has been proposed to play a role in abnormal iron mechanics in endometriosis (Takenaka *et al.*, 2017). In the peritoneal cavity, the vascularization of endometriotic lesions via neo-angiogenesis may be sub-optimal and ectopic lesions are thus likely to be susceptible to high levels of hypoxic stress (Powell *et al.*, 2023). To thrive, lesions need to develop processes such as inflammation, angiogenesis, and steroidogenesis (Hsiao *et al.*, 2015). There is an emerging link between iron physiology and hypoxic conditions (Renassia and Peyssonnaux, 2019), but only one study commented on this topic (Takenaka *et al.*, 2017); therefore, further research is required to clarify the relationship between iron physiology and hypoxia, in the context of endometriosis.

### Systemic iron

Whether localized iron excess translates into abnormal systemic iron metabolism remains unclear. The available studies present contradictory results and primary studies are generally of low quality. Within the published data, there is no convincing evidence of either systemic iron deficiency or excess, except for an association between Stage IV disease and iron deficiency (Hsiao *et al.*, 2015; Kokot *et al.*, 2021). Considering the local haemorrhage into endometriotic lesions (particularly with endometriomas), we can postulate that women with severe endometriosis will lose iron from the circulation and heavy bleeding is also a common complaint in these women. However, it is also possible that this finding relates to excess menstrual losses or dietary insufficiency rather than any generalized metabolic changes in women with endometriosis. It is perhaps unsurprising that systemic iron levels may be unaltered in women with endometriosis, but it is unusual for such a fundamental question to remain without a satisfactory answer. Systemic iron deficiency shares many clinical manifestations with endometriosis, including headache, dizziness or light-headedness, and symptoms of restless leg syndrome (Allen *et al.*, 2013; Tempest *et al.*, 2021). If iron deficiency is confirmed to be prevalent in symptomatic women with endometriosis, iron replacement is a readily available treatment option to alleviate at least some of the symptoms that negatively affect quality of life. Further research is therefore required to delineate both the prevalence of abnormal systemic iron levels and the mechanisms controlling this.

### Iron chelation

If iron excess is accepted to play an important role in initiating and propagating endometriosis, targeting this pathway for potential treatments is an attractive option. Thus far, this has primarily been explored via iron chelation. Iron chelation involves the introduction of an iron-binding compound into an iron-rich environment to bind toxic, free iron into stable iron complexes, rendering it inactive and suitable for recycling or storage. DFO has primarily been utilized within the included studies. DFO has an established role in the clinical management of other diseases characterized by iron excess, including β-thalassaemia (Borgna-Pignatti and Marsella, 2015). In endometriosis, studies are limited to animal models, in which there is surgical induction of an endometriosis-like process in species that do not physiologically menstruate (Defrère *et al.*, 2006; Kizilay *et al.*, 2017). As such, the findings are speculative but intra-peritoneal injections of DFO do demonstrate significant reductions in iron levels, lesion size, and proliferative activity. In theory, a reduction in local iron levels could decrease oxidative stress, inflammation, and lesion proliferation. As such, further research is required to delineate any therapeutic role for iron chelation.

It is also important to examine how current medical therapy, primarily aimed at reducing or stopping menstrual bleeding both from the endometrium, thus reducing retrograde menstruation and locally at the lesion site by manipulating the ovarian cycle, affects local iron overload. This is a further avenue of interest for future study.

Limitations to this review exist, despite the methodological precautions taken throughout. Namely, any review is reliant on the quality of the primary literature. In this case, a minority of the included studies were of objectively low quality with a high risk of bias that may lead to misleading conclusions. Furthermore, multiple studies failed to appropriately characterize the included patients by known confounding variables such as the menstrual cycle phase, which may introduce bias to the findings. In addition, the studies presented significant heterogeneity in patient populations, experimental techniques, and research focus. It is, therefore, challenging to compare their results directly. However, this review provides a contemporary summary of understanding and an overarching viewpoint allowing greater clarity when describing the pathophysiological pathways allowing endometriotic proliferation.

Whether all ectopic endometriosis lesion sub-types go through the same changes and bleed in synchrony with the eutopic endometrium is not yet fully established. The available evidence is limited and typically does not contain matched full-thickness eutopic lesions and different types of ectopic lesions from the same woman with comprehensive assessments of bleeding. This is a further area of study, which will facilitate the understanding of the lesion-specific influence of iron in endometriosis pathogenesis and, thus, the therapeutic significance.

## Conclusions

Degradation of erythrocytes originating from shedding endometrium via retrograde menstruation or ectopic endometriosis lesions leads to localized iron excess in endometriotic lesions. In turn, protective physiological mechanisms are either overwhelmed or primarily defective, allowing toxic iron-mediated oxidative stress to form and maintain a pro-inflammatory environment. Iron excess is associated with and directly impacts endometriotic lesion proliferation, subfertility, symptom severity, and, rarely, malignant transformation. Further research is required, and specific topics highlighted by this review include the role of iron chelation, ferroptosis, the relationship between iron excess and localized hypoxia, systemic iron mechanics in endometriosis, and the role of iron-mediated oxidative stress in malignant transformation.

## Data Availability

The data underlying this article will be shared on reasonable request to the corresponding author.
